# Stigma and Discrimination in People Suffering with a Mood Disorder: A Cross-Sectional Study

**DOI:** 10.1155/2012/724848

**Published:** 2012-04-09

**Authors:** L. Lazowski, M. Koller, H. Stuart, R. Milev

**Affiliations:** ^1^Centre for Neuroscience, Queen's University, 752 King Street West, Postal Bag 603, Kingston, ON, Canada K7L 4X3; ^2^Department of Community Health and Epidemiology, Queen's University, Kingston, ON, Canada K7L 3N6; ^3^Department of Psychiatry, Queen's University, 752 King Street West, Postal Bag 603, Kingston, ON, Canada K7L 4X3

## Abstract

*Background.* Much research is done on the stigma of mental illness, but little research has been done to characterize these phenomena from the perspective of people with mood disorders. *Objective.* To characterize the extent to which individuals with bipolar disorder and depression are stigmatized, determine factors related to higher levels of stigmatization, and assess the reliability of the Inventory of Stigmatizing Experiences in a population of people with a mood disorder. *Methods.* Two hundred and fourteen individuals with depression and bipolar disorder were recruited from a tertiary care psychiatric hospital and surveyed using the Inventory of Stigmatizing Experiences. *Results.* Participants reported high levels of stigma experiences and this did not differ by diagnosis (*P* = 0.578). However, people with bipolar disorder reported greater psychosocial impact of stigma on themselves and their family members compared to people with depression (*P* = 0.019). The two subscales produced internally consistent results with both populations. *Conclusion.* Stigma negatively affects those with both depression and bipolar disorder but appears to have a greater psychosocial impact on those with bipolar disorder.

## 1. Introduction

 The experience of stigma associated with mental illness is devastating and can be detrimental to recovery [1–4]. Link and Phelan [5] defines stigma in terms of five interrelated components: labeling, stereotyping, separation, status loss, and discrimination. They further note that each of these five elements must occur within a power differential, where the stigmatized individual possesses a lesser amount of power. Stigma is often the response to individuals who are expressing an undesirable or frightening characteristic and can be viewed as a continuum from intolerance or agitation to prejudice and discrimination [6, 7]. On the more negative end of the continuum, prejudice and discrimination are rooted in commonly held stereotypes that are associated with mental illnesses. These stereotypes are concentrated within an image that individuals with a mental illness are unable to make competent decisions, are dangerous to themselves and/or the public, and require coercive intervention as they will not seek treatment autonomously [8]. In fact, the diagnosis of a mental illness is coupled with negative stereotypes regardless of the presence of abnormal behavior [9]. Although work has been done to reduce stigma and educate the public about mental illnesses, significant barriers still exist to differentiate people with a mental illness from mainstream society [10].

Social stigmatization of those with mental illnesses has long been studied; however, the majority of analyses have focused on the knowledge and attitudes of the general public. A number of inaccurate ideas about symptoms, etiology and treatments have been identified [11–15]. Social distance is used to measure peoples' willingness to interact with someone who has a mental illness. These studies have shown that many people want to distance themselves from someone who has a mental illness as much as they would with someone with a drug dependency or someone who has been convicted of a crime [12, 16]. 

 Despite the interest in stigma associated with mental illnesses, the perspectives of people with a mental illness have only rarely been studied [17]. Compared to the large body of research assessing public stigma, there has comparatively little work examining the stigma experienced by those with a mental illness, particularly those with a mood disorder [17, 18]. The majority of research has instead focused on stigma within populations of individuals with schizophrenia and other psychotic disorders [17]. However, examining stigma experienced by people with mood disorders is essential as The World Health Organization identifies depression as a major contributor to the global burden of disease due to “its relatively high lifetime prevalence and the significant disability that it causes” [19]. A qualitative study of stigma by Dinos et al. [20] suggests that individuals with mood and anxiety disorders may experience stigma differently when compared to individuals with psychotic disorders highlighting the importance of studying the effects of stigma on individuals with a variety of illnesses.

From the smaller body of research examining stigma from the perspective of someone with a mental illness [18], it has been reported that stigma may greatly affect an individual's decision to seek treatment resulting in delaying or avoiding treatment all together [21, 22]. In those who do seek treatment, stigma may be partially responsible for nonadherence to treatment regimens [1–3]. Perceived stigma has also been shown to be related to reduced social functioning in people with bipolar disorder [23] and impaired functioning in the workplace for people with depression and anxiety [7, 24]. Stigmatization can lead to negative feelings about ones' self-including: shame, self-derogation, feelings of incompetence, and overall low self-esteem [25]. We know of no work that has been done to quantify the degree to which outpatients with mood disorders feel stigmatized and are aware of no studies that have compared the difference in stigma experienced by those with bipolar disorder compared to major depressive disorder.

 Our primary objective was to determine the extent to which people experiencing a mood disorder report being stigmatized. Our secondary objective was to compare stigmatizing experiences of people with a major depressive disorder to those with a bipolar disorder. We hypothesized that having a bipolar disorder would be associated with more stigma than having unipolar depression. We also hypothesized that earlier onset, unemployment, and previous hospitalizations would be associated with greater stigma. A third objective was to further assess the psychometric qualities (specifically, the internal reliability) of the Inventory of Stigmatizing Experiences in a sample of people with mood disorders. The Inventory of Stigmatizing Experiences was developed as a way to measure the impact of stigma and discrimination from the perspective of someone with a mental illness [26]. In previous testing the Inventory scales were found to be highly reliable in a heterogeneous sample with a variety of mental illnesses.

## 2. Methods

**Figure 1:**
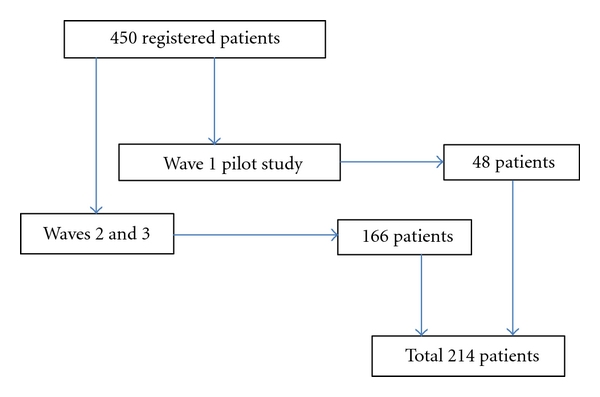
Recruitment of the patient sample.

 This study was approved by the Queen's University Health Science and Affiliated Teaching Hospitals Research Ethics Board. Patients attending a mood disorders outpatient clinic were asked to complete the Inventory of Stigmatizing Experiences as an anonymous questionnaire. As there were no epidemiological studies examining patient reported experiences of stigma among patients with a mood disorder, there were no prevalence data to gauge sample size estimates. Between August 2003 and September 2007, we recruited a convenience sample from the Mood Disorders Research and Treatment Service in Kingston, ON, Canada, in three separate study waves, according to the availability of research funding and support. Two hundred and fourteen clients, out of a total of 450 in the clinic, participated in this research. Forty-eight came from a previous study using this questionnaire [26] (see Figure 1). During the data collection periods, all individuals attending the clinic were invited to participate. Though staff did not formally keep track of refusals, they reported that virtually everyone that was asked did agree to participate. Thus, we estimate that the response rate was at least 80% but probably closer to 90%. There were no restrictions on age, gender, or ethnicity. All participants were registered patients and had received a clinical diagnosis of a mood disorder. Participants were recruited from the intake service and both inpatient and outpatient mood disorders units. All individuals were able to and gave written informed consent before participating. Because individuals who attended the clinic more frequently would have had a greater probability of being recruited, the sample is likely skewed toward clients with more serious disorders and, perhaps, more experiences with stigma. 

### 2.1. Questionnaire

We used the Inventory of Stigmatizing Experiences [26] to assess stigma experiences. Stigma was defined as “negative feelings people have towards people with a mental illness.” The questionnaire consists of a Stigma Experiences Scale (measuring frequency and prevalence) and a Stigma Impact Scale (measuring the intensity of psychosocial impact). This questionnaire has been previously tested for reliability in a heterogeneous sample of psychiatric outpatients. Reliability coefficients were high for both scales: 0.83 for the Stigma Experiences Scale and 0.91 for the Stigma Impact Scale. With respect to scoring, thirteen of the fifteen questions in the Stigma Experiences Scale were answered as “yes,” “unsure” and “no” the other two questions were answered as “never”, “rarely”, “Sometimes”, “Often,” or “Always”. All items were recoded to reflect the presence or absence of stigma (either “yes”, “often” or “always”), with reverse scoring for some items. The Stigma Impact Scale consisted of seven questions, four of which rate the degree to which stigma negatively impacted their individual quality of life, social contacts, family relations, and self-esteem. The remaining three items rated the degree to which stigma negatively impacted their family's quality of life, social contacts, and family relations. Each question was rated on a scale from 0 (lowest possible amount) to 10 (highest possible amount). All responses, including clinical characteristics and health care usage, were self-reported. The questionnaire was administered either as a semistructured interview with a trained member of the research team or as a self-administered survey. The majority of participants completed the questionnaire in interview format. Interviews took place at the clinic or over the phone. Those who choose to fill in the questionnaire themselves were able to complete it in a quiet room or at their homes and then return it.

### 2.2. Statistical Methods

Socioclinical characteristics of the study groups were described using one and two-way frequency distributions with proportions. Internal consistency of the scales was assessed using the Kuder-Richardson (KR 20) reliability coefficient for the experiences scale, which was composed of binary items, and Chronbach's alpha for the impact scale, which was composed of interval data. Item-rest correlations of .40 or less were considered to indicate potentially problematic scale items. These were further assessed by removing them from the scale reliability calculation. If the coefficient of reliability was not substantially improved, then the items were retained. Differences in the stigma experiences of diagnostic groups were assessed using least squares regression with casewise deletion for missing data. Diagnostic group (Major Depression versus Bipolar Disorder) was regressed on each stigma subscale separately with socio-clinical variables added iteratively using a forward entry procedure. Confounding was not evident in any of the models constructed. The final model includes only those variables with statistically significant direct effects. Only participants who answered all scale questions were included in the analyses.

## 3. Results

### 3.1. Sociodemographic and Clinical Characteristics

Table 1 summarizes the socio-clinical characteristics of study subjects by diagnostic group. The majority were female. Ages ranged from 20 to 70, but the majority were between 40 and 59 years. Seventy percent had college or university education, but less than one-third were employed. Approximately half were married and living with a spouse or partner. Less than half considered that their mental health was better now than a year ago, and almost a third thought it was worse. For most, symptoms were first noticed during adolescence or young adulthood. Over a third received treatment within the first year after their symptoms emerged, but a quarter did not receive treatment for more than ten years after their first symptoms. Three quarters had been ill for more than ten years and the majority indicated they had come to accept their diagnosis. Hospital use information was poorly reported with large numbers of missing items—perhaps because this was an outpatient sample with little hospital experience. These questions may have been skipped. We have calculated the valid percents, excluding the missing values. Over a third had been hospitalized in the year prior to the survey, seldom as an involuntary patient. Most (two-thirds) were receiving regular outpatient community mental health treatment. A greater percentage of respondents with Bipolar Disorder were male, 50–59 years, not employed, living alone, previously hospitalized for a mental illness or suicide attempt, and considered their mental health had improved in the previous year. A greater percentage of respondents with depression were university trained, married (or common-law), living with a spouse or partner, and ill less than ten years.

### 3.2. Experience of Stigma

Table 2 summarizes the percent of items endorsed for each of the 10 items comprising the Stigma Experiences Scale for each diagnostic group with reliability coefficients and mean scale scores. All of the scale items were endorsed by a third or more of the respondents and the three items most frequently endorsed (by two-thirds of respondents or more) were the same for each diagnostic group: expectations that the average person would be afraid of someone with a serious mental illness, experiences with stigma having caused them to think less about themselves, and avoiding situations that may be stigmatizing. The item-rest correlations showed that one item was potentially “problematic” but only among people with self-reported depression. In this group, the item measuring the extent to which the average person is thought to be afraid of someone with a mental illness had an item-rest correlation of  0.17. However, Kuder-Richardson coefficients indicated that this sub-scale produced internally consistent data in both groups—well above the minimum conventional cut point of 0.70. The internal consistency of the scale among those with depression was not improved when this item was removed (KR  20 = 0.788) so it was retained for theoretical as well as practical reasons. The mean scale scores were not significantly different, suggesting similar types of stigma experiences occurred within each group. 

### 3.3. Impact of Stigma on Personal and Family Life

Table 3 summarizes the mean item scores and reliability coefficients for the 7-item Stigma Impact Scale. Mean item scores were higher for those with Bipolar Disorder—a difference that was statistically significant (*t*_117df_ = 2.38, *P* = 0.019). Item-rest correlations showed that all items performed well with high alphas for both groups.

Table 4 shows the final results of regression modeling using stigma impact as the dependent variable, and stigma experiences and diagnostic group as the independent variables. Separate models regressing diagnostic group on stigma experiences failed to reach statistical significance. The adjusted *R* ^2^ values for the final model in Table 4 show that the stigma experiences scale was the strongest predictor of stigma impact, accounting for 33.2% of the variance explained. Diagnostic group explained an additional 5.2% of the variance. No other demographic, social, or clinical variables significantly predicted stigma impact. Regression coefficients show that for every point increase on the Stigma Experience Scale (i.e., for every additional stigma experience reported), one would predict a 3.9 point increase in the Stigma Impact Score. In addition, the model predicts that those with a diagnosis of bipolar disorder would have an average scale score 8.96 points higher overall compared to those with depression. Regression diagnostics revealed that assumptions for normality, linearity, homoscedasticity, and collinearity were met. 

## 4. Discussion

This study reports four main findings. First, the two scales used to measure personal experiences with stigma and its psychosocial impacts produce internally consistent results when applied to groups with self-reported depression and bipolar disorder. Reliability coefficients were in the good to excellent range in this sample.

Second, diagnostic group did not differentiate the levels of stigma experienced by subjects, as measured by the Stigma Experiences Scale. This is interesting, as we hypothesized that individuals with Bipolar Disorder would have experienced more stigma. Usually bipolar disorder is more disruptive, and generally more noticeable to the lay public. However, people with depression may be more sensitive and thus report more stigmatizing experiences. In any case this finding requires further study, confirmation with a larger probability sample that will ideally take into account current diagnosis and episode (e.g., depressed versus hypo/manic).

Our third finding was that the diagnostic group did differentiate on the basis of stigma impacts (as measured by the Stigma Impact Scale). Participants with bipolar disorder reported significantly greater psychosocial impact of stigma both for themselves and for their family members. This was an expected finding, as bipolar disorder is considered more severe compared to depression, and more disruptive behaviour is associated with bipolar disorder, which may result in a higher impact of stigma. The reported experiences within the two groups were also unexpectedly similar. We believe that this should be examined more closely with specific attention paid to how the impact may be changing with time, and examining the relationship between the current mental state and degree of stigma impact.

The fourth main finding showed that the frequency of stigmatizing experiences was the strongest predictor of stigma impact, more so than diagnostic group or any other socio-demographic characteristic. One-third of the variance in stigma impact could be explained by the stigmatizing experiences. Additionally, a small but significant amount of variance (5.2%) was explained by diagnostic group. All other sociodemographic or clinical characteristics did not prove to be predictive in this sample. This is a particularly important finding as far as our understanding of illness stigma is concerned. This suggests that if we are able to influence the frequency of stigmatizing experiences, through antistigma programs, we may be able to reduce the impact as well.

Past-population-based research has shown that members of the general public hold different stereotypes for different diagnostic groups. In a large multinational study Pescosolido and colleagues [8] reported that people in the general public, regardless of the social situation, are more likely to distance themselves from people with schizophrenia than people with depression. This research would suggest that stigma experiences should differ depending on diagnosis. However, our results suggest that the public may not make diagnostic distinctions when they interact with people with a mental illness; at least with respect to distinctions between those with depression and bipolar disorder. In our research, people with major depression and bipolar disorder described a similar range of stigma experiences.

Not only are the stigmatizing experiences of individuals who have a mental illness important, but also the psychosocial impact of these experiences is equally important to quantify and understand. The stigma impact scale revealed that individuals with bipolar disorder felt more psychosocial impact from their experiences with stigma than did people with depression. As well, they reported a more negative impact on their family members. Anecdotally, in the community, people with bipolar disorder experience more self-stigma, especially related to portrayals in the media, and this may be related to the greater impact felt from their stigmatizing experiences. Results from this study highlight the importance of differentiating between the type and frequency of stigma experiences that are reported by people with a mental illness and their psychosocial impacts.

Stigma due to mental illness is a pervasive phenomenon. This is seen in the high level of endorsement of items in the stigmatizing experiences scale. Seven of the ten items of the stigma experiences scale were endorsed by at least 50% by both individuals with depression and bipolar disorder, with peak endorsement reaching 73.3%.

The relationship between the frequency of stigmatizing experiences and their impact has not been fully elucidated. We found that one-third of the impact of stigma experienced by our sample subjects was explained by stigma experiences, and another 5% was explained by diagnosis. Given that the model explained just over a third of the variation, there are many additional factors contributing to the impact of stigma that still remain to be discovered. An interesting future direction would be to examine the extent to which culture modifies the impact of stigmatizing experiences on psychosocial domains. Investigation into attributes, resilience factors, and social supports, which may protect individuals from the negative impact of stigma, also remains to be explored. The Inventory of Stigmatizing Experiences represents one tool needed to fully characterize the effects of stigma felt by people suffering mood disorders as well as other mental illnesses. Now that the internal consistency of the scales has been demonstrated in both heterogeneous and homogeneous clinic populations, future research is needed to assess the validity of these scales: to determine whether they are associated with other stigma-related constructs (such as self-esteem or empowerment) in predictable ways and to determine whether they predict clinical outcomes such as recovery, medication adherence, or relapse. It will also be important to determine if the scale scores are sensitive to change so that they could be used to evaluate anti stigma interventions.

One limitation of the study is that participants were recruited from a tertiary referral university centre and may represent a more severely ill and chronic population. Individuals who attended the clinic more frequently would have had a greater probability of being included in our convenience sample. There may also be a bias toward older individuals for the same reason. While this should not affect our ability to assess the internal consistency of the scales, the impact of selection bias on our descriptive findings is difficult to evaluate. On the one hand, people with more chronic conditions may perceive a higher degree of stigma, but on the other, they may be able to deal better with the impact due to the fact that they have been in treatment for some time. In future studies, it will be important to ensure a more representative sampling in order to evaluate the nature of illness severity and duration on stigma experiences and impact. Also, as our study reflects patients recruited between 2003 and 2007, we need to address the time changes with a future study.

In conclusion, it is important to assess stigma experiences and their impact from the perspective of people who are affected by mental illness. Research in this area has been hampered by the lack of psychometrically tested scales. This research has demonstrated that the Inventory of Stigma Experiences, which was originally developed for use in a heterogeneous sample of outpatients, also produces reliable results in a diagnostically homogeneous sample. Participants reported a high degree of stigmatizing experiences and the extent of experiences was similar in depression and bipolar disorder.

## Figures and Tables

**Table 1:** Social characteristics of participants (*N* = 190)*.

Characteristic	Bipolar disorder % (*N*)	Depression % (*N*)	Total % (*N*)
Gender			
Male	41.7% (35)	33.3% (35)	37.0% (70)
Female	58.3% (49)	66.7% (70)	63.0% (119)

Age group			
20–29	8.4% (7)	10.7% (11)	9.7% (18)
30–39	15.7% (13)	15.5% (16)	15.6% (29)
40–49	33.7% (28)	35.0% (36)	34.4% (64)
50–59	37.3% (31)	30.1% (31)	33.3% (62)
60–70	4.8% (4)	8.7% (9)	7.0% (13)

Highest education			
Public school or less	2.4% (2)	2.9% (3)	2.6% (5)
High school	29.8% (25)	24.8% (26)	27.0% (51)
College or technical training	39.3% (33)	37.1% (39)	38.1% (72)
University	28.6% (24)	35.2% (37)	32.3% (61)

Employment status			
Employed	27.4% (23)	36.2% (38)	32.3% (61)
Not employed	72.6% (61)	63.8% (67)	67.7% (128)

Marital status			
Single	56.0% (47)	47.6% (50)	51.3% (97)
Married/common law	44.0% (37)	52.4% (55)	48.7% (92)

Living situation			
Alone	33.3% (28)	25.7% (27)	29.1% (55)
Spouse/partner	44.0% (37)	50.5% (53)	47.6% (90)
Parents	6.0% (5)	9.5% (10)	7.9% (15)
Other	16.7% (14)	14.3% (15)	15.3% (29)

Mental health now compared to a year ago			
Better	52.4% (44)	34.3% (36)	42.3% (80)
About the same	23.8% (20)	30.5% (32)	27.5% (52)
Worse	23.8% (20)	35.2% (37)	30.2% (57)

Age that symptoms were first noticed			
10 or under	8.2% (7)	6.0% (6)	7.0% (13)
11–19	41.2% (35)	38.0% (38)	39.5% (73)
20–29	20.0% (17)	21.0% (21)	20.5% (38)
30–39	21.2% (18)	22.0% (22)	21.6% (40)
40+	9.4% (8)	13.0% (13)	11.4% (21)

Number of years ill (as of survey completion)			
10 or less	22.9% (19)	30.6% (30)	27.1% (49)
11–19	22.9% (19)	21.4% (21)	22.1% (40)
20–29	18.1% (15)	22.4% (22)	20.4% (37)
30–39	22.9% (19)	19.4% (19)	21.0% (38)
40–51	13.3% (11)	6.1% (6)	9.4% (17)

Age at first treatment			
2–19	22.5% (18)	16.8% (16)	19.4% (34)
20–29	25.0% (20)	30.5% (29)	28.0% (49)
30–39	30.0% (24)	28.4% (27)	29.1% (51)
40+	22.5% (18)	24.2% (23)	23.4% (41)

Number of years between symptoms and first treatment			
Under 1 year	38.8% (31)	39.6% (36)	39.2% (67)
1-2 years	11.3% (9)	9.9% (9)	10.5% (18)
3–5 years	16.3% (13)	14.3% (13)	15.2% (26)
6–10 years	10.0% (8)	8.8% (8)	9.4% (16)
10+ years	23.8% (19)	27.5% (25)	25.7% (44)

Have come to accept diagnosis			
No	17.6% (15)	14.6% (15)	16.0% (30)
Yes	82.4% (70)	85.4% (88)	84.0% (158)

Years between treatment initiation and diagnosis acceptance			
Not accepted	19.2% (15)	18.1% (15)	18.6% (30)
Less than 1 year	23.1% (18)	26.5% (22)	24.8% (40)
1–5	25.6% (20)	24.1% (20)	24.8% (40)
6–10	11.5% (9)	15.7% (13)	13.7% (22)
11–15	7.7% (6)	7.2% (6)	7.5% (12)
16–20	5.1% (4)	4.8% (4)	5.0% (8)
21–25	7.7% (6)	3.6% (3)	5.6% (9)

Ever hospitalized for a mental illness or suicide attempt			
Yes	65.9% (56)	53.8% (56)	59.3% (112)
No	34.1% (29)	46.2% (48)	40.7% (77)
Unknown/missing	(0)	(1)	(1)

Ever hospitalized in a provincial psychiatric institution			
Yes	55.4% (31)	52.7% (29)	54.1% (60)
No	44.6% (25)	47.3% (26)	45.9% (51)
Unknown/missing	(29)	(50)	(79)

Ever hospitalized in a general hospital psychiatric unit			
Yes	78.6% (44)	67.3% (37)	73.0% (81)
No	21.4% (12)	32.7% (18)	27.0% (30)
Unknown	(29)	(50)	(79)

Ever committed under provincial mental health legislation			
Yes	38.2% (21)	11.1% (6)	24.8% (27)
No	61.8% (34)	88.9% (48)	75.2% (82)
Unknown/missing	(30)	(51)	(81)

Ever remanded to a forensic unit under federal legislation			
Yes	4.9% (2)	0.0% (0)	2.7% (2)
No	95.1% (39)	100.0% (33)	97.3% (72)
Unknown/missing	(44)	(72)	(116)

Hospitalized as a voluntary patient in the last year			
Yes	30.9% (17)	40.0% (22)	35.5% (39)
No	69.1% (38)	60.0% (33)	64.5% (71)
Unknown/missing	(30)	(50)	(80)

Hospitalized as an involuntary patient in the last year			
Yes	3.6% (2)	3.6% (2)	3.6% (4)
No	69.1% (38)	60.0% (33)	64.5% (71)
Unknown/missing	(30)	(50)	(80)

Use of outpatient community mental health program in the last year			
Yes	64.7% (55)	68.3% (71)	66.7% (126)
No	35.3% (30)	31.7% (33)	33.3% (63)
Unknown/Missing	(0)	(1)	(1)

Frequency of outpatient treatment (*N* = 124)			
Weekly	46.3% (25)	41.4% (29)	43.5% (54)
2-3 times per month	9.3% (5)	24.3% (17)	17.7% (22)
Monthly	22.2% (12)	21.4% (15)	21.8% (27)
Every 2-3 months	14.8% (8)	10.0% (7)	12.1% (15)
1-2 per year	7.4% (4)	1.9% (2)	4.8% (6)

*Missing data for some items will mean that frequencies may not total.

**Table 2:** Reliability coefficients for the 10-item Stigma Experience Scale.

Scale item	Bipolar Disorder (*N* = 79*)		Depression (*N* = 90*)	
	% Endorsed	Item-rest correlation	% Endorsed	Item-rest correlation
Do you think people will think less of you if they know you have a mental illness?	62.0%	.32	61.1%	.35

Do you think that the average person is afraid of someone with a serious mental illness?	69.6%	.46	73.3%	.17

Have you ever been teased, bullied, or harassed because you have a mental illness?	43.0%	.44	36.7%	.54

Have you felt that you have been treated unfairly or that your rights have been denied because you have a mental illness?	53.2%	.32	50.0%	.49

Have your experiences with stigma affected your recovery?	51.9%	.46	56.7%	.60

Have your experiences with stigma caused you to think less about yourself or your abilities?	65.8%	.45	71.1%	.49

Have your experiences with stigma affected your ability to make or keep friends?	49.4%	.58	55.6%	.45

Have your experiences with stigma affected your ability to interact with your family?	55.7%	.45	55.6%	.42

Have your experiences with stigma affected your satisfaction with or quality of life?	60.8%	.48	70.0%	.45

Do you try to avoid situations that may be stigmatizing to you?	65.8%	.40	71.1%	.45

Kuder-Richarson coefficient of reliability (KR-20)		**.77**		**.78**

Mean Scale Score (SD)**		**5.8 (2.8)**		**6.0 (2.8)**

CI		**5.1–6.4**		**5.4–6.6**

*Note reduced sample size. Only respondents who answered all scale questions are included in the above table. **t  (167 df) =  .558, *P* = .578.

**Table 3:** Reliability coefficients for the 7-item Stigma Impact Scale.

Scale item	Bipolar Disorder (*N *= 57*)		Depression (*N* = 62*)	
	Mean (SD)	Item-rest correlation	Mean (SD)	Item-rest correlation
On a scale where 0 is the lowest possible amount, and 10 is the highest possible amount, how much has stigma affected *you personally*?				
Quality of life	5.6 (2.8)	.67	4.7 (3.0)	.72
Social contacts	5.9 (3.3)	.70	5.3 (3.1)	.74
Family relations	5.2 (3.5)	.56	3.7 (3.2)	.75
Self-esteem	6.2 (3.1)	.47	6.0 (3.3)	.67

On a scale where 0 is the lowest possible amount, and 10 is the highest possible amount, how much has stigma affected *your family as a whole*?				
Quality of Life	4.8 (3.8)	.77	3.3 (3.3)	.75
Social Contacts	4.6 (3.8)	.82	3.2 (3.1)	.76
Family relations	5.3 (3.2)	.77	3.3 (3.2)	.76

Chronbach's Alpha reliability coefficient		**.89**		**.91**

Mean scale score (SD)**		**37.5 (18.3)**		**29.5 (18.1)**

CI		**32.6–42.3**		**24.9–34.1**

*Note reduced sample size. Only respondents who answered all scale questions are included in the above table. **t  (117 df) = 2.38, *P* = 0.019.

**Table 4:** Final model summary.

Model	Coefficients	*R * ^2^ change	*F* statistic (for change)	Significance of *F* change
Average (constant)	5.95			
+ Stigma Experiences Scale Score	3.96	.338	55.135_(df = 1,108)_	<.001
+ Diagnostic Group	8.96	.058	10.228_(df = 1,107)_	<.001

Stigma impact score = 5.95 + 3.93*x* (stigma experience score) + 8.96*x* (0 = depression; 1 = bipolar).
